# Precision Therapeutics Through Bioactive Compounds: Metabolic Reprogramming, Omics Integration, and Drug Repurposing Strategies

**DOI:** 10.3390/ijms262010047

**Published:** 2025-10-15

**Authors:** Michele Costanzo, Giovanni N. Roviello

**Affiliations:** 1 Department of Molecular Medicine and Medical Biotechnology, University of Naples Federico II, 80131 Naples, Italy; 2 CEINGE—Biotecnologie Avanzate Franco Salvatore, 80145 Naples, Italy; 3 CNR Institute of Biostructures and Bioimaging, Via Tommaso De Amicis 95, 80145 Naples, Italy

Bioactive compounds, whether derived from nature or synthetically produced, continue to redefine the landscape of therapeutic innovation. Integrating bioactive compounds with omics technologies [1,2] and metabolic profiling [3] unlocks transformative insights for precision medicine [4], drug repurposing [5], and systems-level therapeutic innovation. Remarkably, the pursuit of cutting-edge therapies for socially impactful diseases, especially neurodegenerative pathologies [6,7], centers on molecular platforms that integrate both natural and synthetic compounds, ranging from phytomolecules, amino acids, and peptides to synthetic heteroaromatic compounds [8,9,10,11]. Their ability to modulate molecular pathways, restore metabolic balance, and interact with complex biological systems positions them as key agents in the interest of precision medicine. The Topic “Bioactive Compounds and Therapeutics: Molecular Aspects, Metabolic Profiles, and Omics Studies” (https://www.mdpi.com/topics/LL4H8331Z3 accessed on 1 October 2025) brings together a diverse collection of 23 studies that explore the therapeutic potential of bioactive molecules through the lens of molecular biology, metabolic profiling, and omics technologies (Table 1). Natural products such as ursolic acid (Figure 1), rosemary extract, and hemp seed oil demonstrate promising effects on immune regulation, lipid metabolism, and adipogenesis [12,13].

Synthetic derivatives like halogenated boroxine [14] and novel quinazoline compounds reveal targeted actions on cancer cell autophagy and skin barrier function. The repositioning of existing drugs, exemplified by moxidectin’s antimalarial activity and GHF-201′s autophagic activation [15] in glycogenosis, underscores the value of drug repurposing in rare and infectious diseases. Several studies delve into the gut–brain axis [16,17,18,19,20,21,22], microbiota modulation, and metabolic syndrome, highlighting the systemic impact of bioactive agents. For instance, dietary methionine restriction [23] and algal fiber-rich formulas show metabolic improvements in animal models, while GPR40/GPR120 agonists [24] alleviate inflammation-linked periodontitis. The integration of omics approaches, such as glycan microarrays [25,26,27,28], metabolomic signatures in colitis [29], and proteomic insights into antibody secretion [30], provides a systems-level understanding of therapeutic mechanisms in some disease conditions. Research into bioactive compounds continues to evolve beyond traditional boundaries, encompassing not only human health but also environmental and structural biology. In particular, investigations into plant stress responses [31,32,33], telomere biology [34,35], and drug pharmacokinetics [36,37,38,39,40,41] exemplify this expanded scope, revealing how bioactivity influences diverse biological systems across species and contexts. These studies underscore the interconnectedness of molecular mechanisms that govern resilience, aging, and therapeutic efficacy.

Together, these insights illuminate the path from molecular understanding to clinical relevance by bridging bioactivity with omics technologies and metabolic profiling, thereby fostering a deeper understanding of how therapeutic agents can be harnessed for targeted, effective, and personalized interventions across a wide spectrum of biological systems, disease models, and therapeutic contexts, ultimately advancing precision medicine through integrative approaches that combine molecular characterization, functional analysis, and translational application.

## Figures and Tables

**Figure 1:**
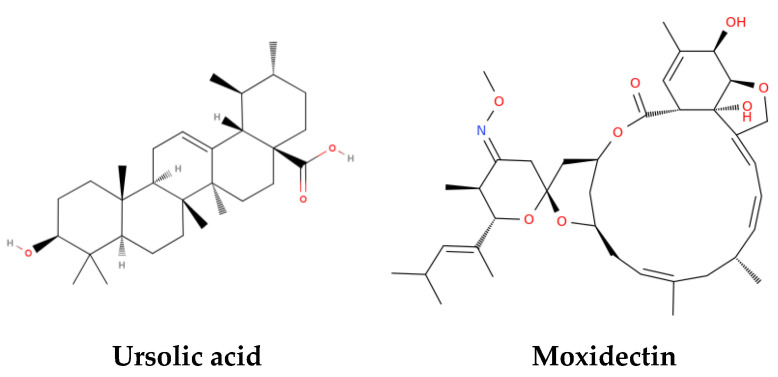
Structural representations of ursolic acid and moxidectin [12,13]. Moxidectin is a macrocyclic lactone with antiparasitic activity. Ursolic acid is a triterpenoid known for its anti-inflammatory and anticancer effects.

**Table 1:** Summary of the research collection on bioactive compounds.

Category	Examples	Therapeutic Focus
Natural Products	Ursolic acid, rosemary extract, hemp seed oil	Immune regulation, lipid metabolism, adipogenesis
Synthetic Derivatives	Halogenated boroxine, quinazoline compounds	Cancer cell autophagy, skin barrier function
Drug Repurposing	Moxidectin, GHF-201	Antimalarial activity, autophagic activation in glycogenosis
Metabolic Modulators	Methionine restriction, algal fiber-rich formulas, GPR40/GPR120 agonists	Gut health, metabolic syndrome, inflammation-linked periodontitis
Omics-Based Approaches	Glycan microarrays, metabolomic and proteomic signatures	Systems-level understanding of therapeutic mechanisms
Other Investigations	Plant stress responses, telomere biology, drug pharmacokinetics	Environmental biology, structural biology, molecular profiling
Methodologies Used	In vitro assays, in vivo models, in silico simulations	Multidisciplinary exploration from molecular insight to clinical relevance

## Data Availability

Not applicable.
